# The medical licensing assessment (MLA) content map: A list is not a syllabus, and a syllabus is not a curriculum

**DOI:** 10.1016/j.clinme.2025.100311

**Published:** 2025-04-02

**Authors:** Isobel Walker, Rakesh Patel

**Affiliations:** a Epsom and St Helier University Hospitals NHS Trust, Wrythe Lane, Carshalton, Surrey, SM5 1AA, United Kingdom; b Barts and the London School of Medicine, Garrod Building, Turner St, London, E1 2AD, United Kingdom

**Keywords:** Medical education, Medical licensing assessment, Undergraduate, Curriculum design, Syllabus, Collaboration

## Abstract

The GMC’s Medical Licensing Assessment (MLA) marks a pivotal moment in UK medical education: the 2025 graduating cohort will be the first required to pass this national exam before entering clinical practice. The accompanying MLA ‘Content Map’ lists 217 clinical presentations and 315 conditions – across 25 areas of clinical practice that students need to know by the end of the programmes. While some medical educators may simply use this list to confirm whether timetabled teaching covers the material required, others may use the list a driver for curriculum change and transformation. In the case of the latter, meaningful opportunity now exists for collaborative redesign of undergraduate programmes, comprising everything from removing terms such as ‘pre-clinical’ and ‘clinical’ through to promoting more integrated approaches to delivering medical education and developing clinical reasoning using more authentic, applied workplace-based learning activities.

## Introduction

The General Medical Council’s (GMC) Medical Licensing Assessment (MLA)^1^ has captured the attention of all medical students in the UK, since the 2024–2025 cohort will become the first required to sit and pass a national exam before they are allowed to enter clinical practice. In order to support students to prepare for the MLA, the GMC has published a ‘content map’,^2^ which lists 217 presentations and 315 conditions that items will cover on the Applied Knowledge Test (AKT) (two 100-question single-best answer (SBA) assessments) and stations in the Clinical and Professional Skills Assessment (CPSA) that comprise the MLA. The clinical presentations are initially listed in alphabetical order, but they are also organised by condition and clinical subspecialty in the document, and comprise the subject-matter domain of content knowledge that students would be expected to know at the end of their undergraduate training. All medical schools will now have to ensure sufficient coverage of these clinical presentations in their undergraduate curricula to reassure both students but also stakeholders such as the GMC and the public.

However, the challenge for many medical schools, particularly those with established programmes of study, is both philosophical and practical divide of ‘pre-clinical’ and ‘clinical’ subject-matter domain knowledge: the former being traditionally taught in classrooms settings in the early years, and the latter generally taught in authentic clinical workplace environments.^3^ As a consequence, the way in which teaching content is currently delivered can lack relevance for students in the early years, and students may also fail to remember the importance of fundamental concepts or principles as they relate to clinical presentations in the later years.^4^ Similarly, the way in which content is revisited in both early and later years may be inconsistent and lack coherence for students as they receive teaching from multiple teachers over the length of their course.

There is now an opportunity for all medical educators to review their undergraduate curricula and re-orientate the teaching of subject-matter domain knowledge so that there is explicit alignment with the content map, but also work with students to reform or redesign their curricula altogether to ensure that their learning experience continues to provide a rich, and broad education. A simplistic approach to using the content map would be to merely cross-reference the 217 presentations and 315 conditions to existing timetabled activity, whereas a sophisticated approach could be re-look at the way in which the same presentation could be taught or learnt in different care contexts, or the same condition could be taught by the different clinical subspecialties.

The purpose of this paper is to share some insights from the wider medical education literature, as well as personal and practical experience from curriculum development efforts, in order to avoid a situation whereby educators only use the list of clinical presentations as a mapping tool for cross-referencing their teaching with written questions tested in the end-of-year or end-of-course assessments across medical schools. These theory-driven insights can enable medical educators to use the content map as a driver for redesigning curricula and ensuring that the development of students’ professional knowledge continues to happen in parallel with the development of professional skills, behaviours and attitudes necessary for students to also safely enter practice at the end of their undergraduate training. The insights are also based on core evidence-based educational concepts to make classroom teaching more effective, and include reflections over common misconceptions around student learning, to avoid inefficient teaching delivery while on placement.

### The MLA content map, a syllabus and a curriculum

The MLA is a two-part exam that effectively assesses whether a student seeking entry to the UK medical register can practise safely. The AKT is set ‘centrally’ by the Medical Schools Council, with the quality assurance function performed by the GMC.^5^ The AKT comprises two online 100-question examinations in a single best answer (SBA) format, and tests students’ application of subject-matter domain content knowledge across different clinical scenarios. The CPSA comprises the practical aspect of the MLA, but is hosted ‘locally’ by individual medical schools and tests both the application of knowledge, but also the skills and behaviours demonstrated by students across different context-specific clinical situations.

**Fig. 1:**
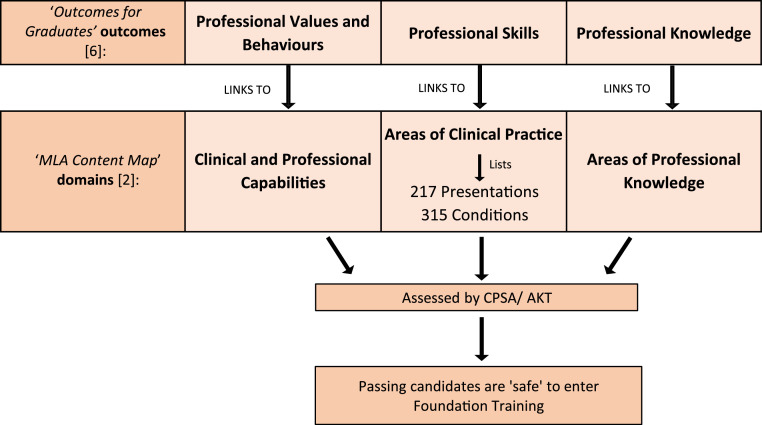
A schematic demonstrating how *outcomes for graduates* links to the *MLA content map*, and student pathways if educators use the document as a syllabus.

The GMC’s MLA content map, upon which items in the AKT and CPSA are designed, is split into three domains which directly link to GMC oOutcomes for graduates’^6^ (Fig. 1) – ‘areas of clinical practice’, ‘areas of professional knowledge’ and ‘clinical and professional capabilities’. The 217 presentations and 315 conditions all fall under 25 ‘areas of clinical practice’. The GMC defines the presentations as: ‘signs, symptoms, investigation results and other relevant patient-related issues’; and conditions as ‘pathophysiological diseases or clinical diagnoses’.^2^ The decision around what gets included in the content map is made based on the presentations and conditions that graduating doctors are most likely to encounter in their foundation training programme.

The first misconception to avoid is not conflating this content map, which is effectively just a list of either presentations or conditions, with a syllabus. Put another way, a content map is not a syllabus in and of itself, but rather a starting point from which the latter could be developed and become a broader framework for organising any teaching about clinical presentations. A syllabus provides a detailed outline of the material that a course comprises, and also includes information about how students will be taught and assessed.^7^ Therefore, any MLA syllabus for undergraduate medicine would need that additional information around teaching delivery, including a full description of what content and how an educator teaches it, both of which would likely vary across different medical schools. Medical schools will now need to make this information explicit for their individual programmes, and produce an output that could constitute a MLA syllabus of sorts, that drives both students’ learning but also directs clinicians’ teaching; rather than just proceed with using the existing MLA content map on its own, which contains none of this information, nor is it ever likely to.

A syllabus is also not the same as a curriculum.^8^ While a syllabus forms an integral part of a curriculum, the curriculum in and of itself is broader than a single document, and essentially constitutes the entire organising system of education developed to support students to achieve their learning outcomes. The curriculum encompasses not only all the teaching within a programme, but also the assessment methods, all the educational activities and the ways in which educators create the lived experience for students. Therefore, within a curriculum, different teaching syllabi (eg anatomy, physiology, clinical skills, patient safety, leadership) align with the learning objectives and learning outcomes for the programme of study. The curriculum in medical education contexts is generally the shared responsibility of more than one educational provider: primarily the medical school in the early years, but also a variety of NHS providers in primary and secondary care during the later years.

**Fig. 2:**
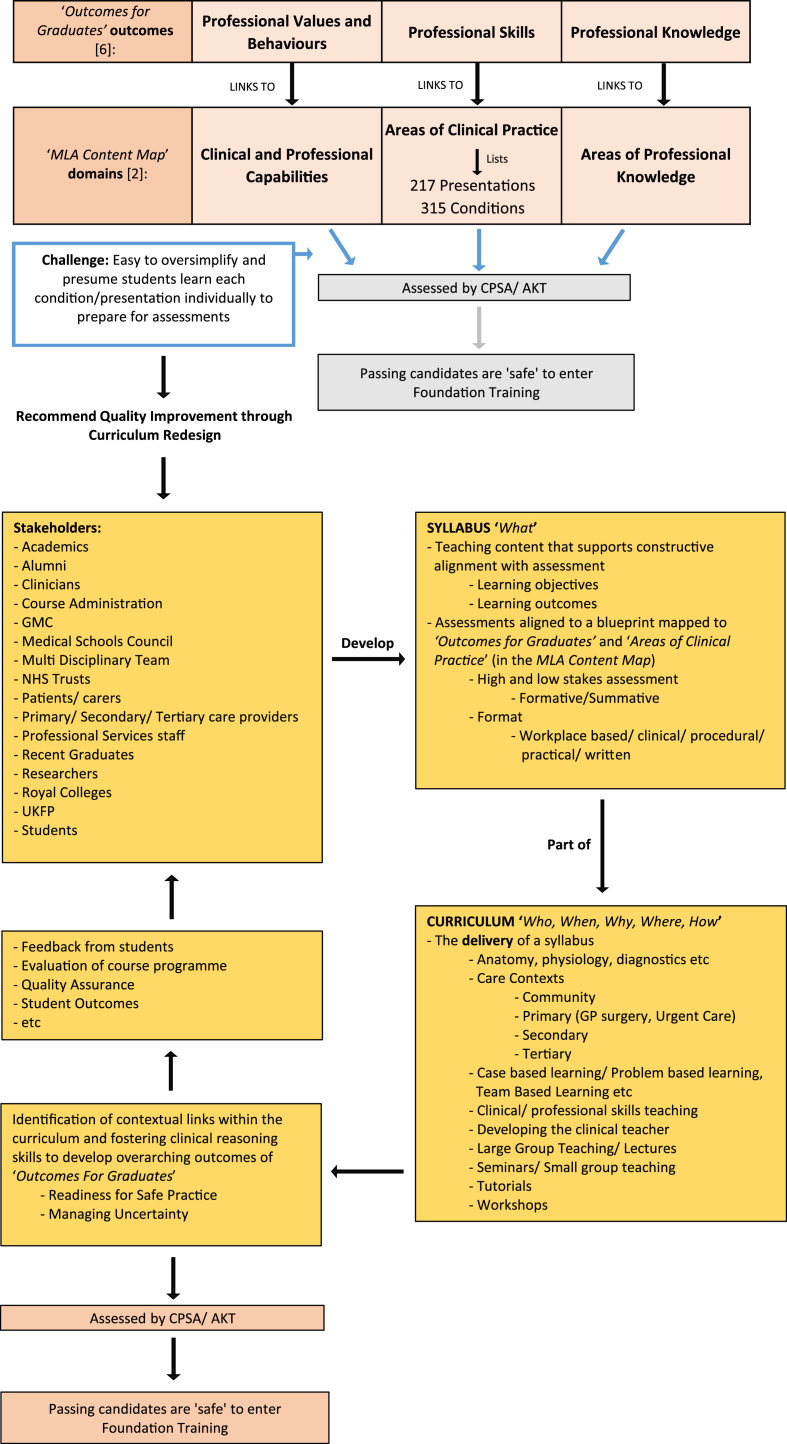
A schematic demonstrating how *outcomes for graduates* links to the *MLA content map*, but where student pathways are designed beyond the document and are *part of* a wider curriculum transformation process.

The real opportunity now for medical educators is to use the MLA content map to develop syllabi integrating all the MLA presentations and conditions across undergraduate training programmes, but also use it as a trigger for reflecting over how far and wide educators need to go in their coverage of this content. Likewise, the student experience will not be determined by the MLA content map or any accompanying syllabus, but rather the way in which curriculum activities are planned by the medical school, and then come together in a coherent manner when implemented by placement providers across the whole programme. The content map can therefore also serve as a catalyst for medical schools and NHS partners to work collaboratively in order to review and redesign the curriculum together, delivering both improvements in teaching content and formative learning opportunities for students in the process (Fig. 2).

### Teaching about presentations and conditions

The traditional way that most medical curricula are designed involves organising teaching about the fundamental or pure sciences in the early years, (eg anatomy, biochemistry, immunology, physiology, pathology, pharmacology) and organising teaching around the clinical sciences in the later years (eg anaesthetics, cardiology, gastroenterology, neurology, musculoskeletal, paediatrics, respiratory, surgery). Furthermore, within medical curricula, clinical presentations are used for different purposes depending on whether these are being taught in the early years or later years. For example, diabetic ketoacidosis in the early years may be used to make explicit the relevance of a scientific concept such as the Krebs cycle,^9^ whereas in the later years, the same presentation may be used for highlighting the use of intravenous fluids, or the importance of knowing the different between fixed- or variable-rate insulin infusions from a patient safety perspective.

A well-designed curriculum could ensure that the scientific basis of different conditions is learnt in the early years, ensuring students are able to reason through the clinical presentations across different care contexts, and acknowledge subspecialty-specific differences in the later years as they follow patients through the healthcare system. Only using the MLA content map as a simple list of presentations or conditions may not enable medical educators to design placement experiences in way that effectively achieves this level of desired outcome. Furthermore, medical educators who use the MLA content map as separate lists of presentations and conditions only risk students not appreciating the way in which the two integrate in clinical practice, since students lack experience in the workplace to make these learning links. Likewise, using the MLA content map as a list in and of itself also risks students not perceiving the need to understand the underlying fundamental sciences, or failing to recall these links when applying them to clinical practice in workplace settings.^10^ Given that the content map underpins the MLA, there is also a concern that the lists imply to students that simply recalling facts is sufficient for passing the exam, and that being a doctor merely involves picking a single-best answer for a given presentation or condition from a pre-defined choice of options.

As all curriculum designers review their educational offer to students in the MLA era, medical educators could work more closely with clinical teachers to write learning objectives and outcomes to mitigate these risks. While recalling knowledge is important, the correct application of knowledge at the point of care is most important in medical education. The challenge for students, who lack clinical experience, is to apply knowledge and skills to a given clinical problem when both the presentation may be ambiguous to them, and the condition or conditions responsible, also remain uncertain for them. By ensuring that the teaching of clinical presentations and conditions is integrated, as well as replicated over a programme, students’ learning will improve. While duplication of teaching content is less desirable,^11^ repetition of core or difficult concepts is a good thing: increased student learning happens when educators build on what is already known by students, and students themselves are actively encouraged to recall that knowledge in practice.^12^ By designing curricula together, clinicians in practice alongside academic medical educators can better plan learning more effectively, by ensuring that teaching is stage appropriate for the development of the learner, rather than based on what expertise or interest the teacher has in a given clinical condition.

### Clinical reasoning, patient safety and outcomes for graduates

Some of the reasons for implementing the national MLA include ensuring that students achieve the professional knowledge, skills and behaviours outlined in the GMC’s Outcomes for Graduates, and that graduates are adequately prepared to enter foundation training to perform patient safety-critical workplace-based tasks involving clinical decision making and prescribing. Underpinning both tasks is clinical reasoning, which is the ability to make a diagnosis and propose a management plan when delivering care to patients.^13^ The challenge with developing clinical reasoning expertise in the early years of undergraduate training is ensuring that: 1) students build sufficient knowledge in the fundamental, social and psychological sciences, 2) the sufficient number of mental representations necessary for solving clinical problems are constructed when they encounter clinical presentations in practice.

Likewise, the challenges in the later years of undergraduate training are: 1) exposing students to enough patients across different care contexts so that they can practise recalling this knowledge, 2) applying it to authentic patient presentations across the spectrum of health and disease states, and 3) evolving their mental representations into illness scripts or schema that acknowledge broader concepts such as patient safety, complexity and uncertainty, as well as safe-guarding, leadership, team-working and delivering patient-centred care. For this knowledge to be both secure over time, and transferred by the individual from one clinical encounter to the next, there needs to be feedback given by clinicians who have the experience and expertise in different workplace environments.

The MLA content map, while making explicit the 217 presentations and 315 conditions across which items in the MLA will be drawn from, may also be inadvertently used by students as a list of revision labels to be learnt away from the bedside, eg in a library and distant from authentic clinical environments where this contextual knowledge is situated. While there is strong evidence that the more questions students practise, the more likely that their performance on written examinations will be better,^14^ evidence for the effectiveness of this strategy with respect to increasing clinical reasoning performance in practice is lacking. If anything, clinical reasoning performance is not consistent across different cases and different situational contexts in the real world, with a significant proportion of the variance in performance within the same individual likely to do with variables related to the patient or the care encounter.^15^

In order to develop clinical reasoning expertise across an undergraduate medical curriculum, students need to practice history-taking, physical examination, requesting and interpreting tests across many hundreds of cases in authentic workplace-based settings. Therefore, there is a real opportunity to use evidence-based and theory-driven approaches to design and develop undergraduate curricula using the MLA content map as a driver for change, encouraging students away from exclusively studying in library-type environments. In particular, the complexity of clinical practice can and should be foregrounded in the design of placement learning experiences by using presentations, cross-linked with conditions, delivered by a multiple clinical subspecialties to represent the way in which clinical reasoning actually happens in the workplace. Rarely do patients have a single problem resulting from a given clinical presentation, or they are rarely cared for by a single subspecialty team. For the first time, medical educators across the field from undergraduate educators through to NHS providers can collaborate around a single document and design activities that are both relevant and important as learning experiences, but also necessary for achieving programme-level learning outcomes.

The list of presentations in the MLA content map can be used to create a sophisticated clinical reasoning learning journey through medicine, by identifying which conditions are most associated with them in terms of incidence and prevalence. Furthermore, the list of presentations linked to conditions can then be used to identify within which care contexts students might encounter them, and which clinical subspecialties should collaborate together when delivering teaching to achieve optimal outcomes (Fig. 2). The traditional approach of designating a list of conditions to a single or primary clinical subspecialty to teach alone seems redundant in the era of an aging, multimorbid population with complex care needs.^16^ Likewise, some presentations can be associated with multiple conditions, and some conditions with multiple presentations require a multidisciplinary team approach to delivering teaching. It should also be remembered that individual presentations and conditions may still require further consideration, since paediatric and adult medicine remain distinct. Therefore, the MLA content map can drive more coherence, consistency and comprehension of content for students, by inviting complementary clinical subspecialties to work together in a systematic way across a medical degree.

## Conclusion

The introduction of the Medical Licensing Assessment (MLA) represents a transformative opportunity for medical education in the UK, despite some very valid concerns from both medical educators and students alike about its purpose and impact on training. This is because, for the first time, the MLA content map now provides a single point of reference about the subject domain from which items in the AKT and CPSA will be drawn. However, moving beyond the prism of assessment and looking more broadly at teaching and learning, the MLA also provides medical educators with a powerful driver and catalyst for fundamental curriculum redesign.

The traditional perceived, rather than real or actual, division within undergraduate medical education – between pre-clinical and clinical learning – can properly be challenged as well as reimagined, since both content and contextual knowledge need to be acquired by students preparing for the MLA across the whole programme of study. Likewise, the ownership of teaching the content on the map lies among all education providers, hence the resource can also drive systemic collaboration across those responsible for both early and later years medical training in the UK. This collaborative approach is essential if medical students are to enter foundation training able to navigate the complexities of healthcare in the NHS, characterised by multimorbidity, an ageing population and interdisciplinary care.

Finally, medical educators now have the opportunity to move beyond simplistic cross-referencing of existing curricula with the MLA content map, but rather use the resource to develop sophisticated, holistic learning experiences that explicitly connect fundamental scientific principles taught in the classroom with the real-world challenge of performing clinical reasoning in the workplace. As medical education enters this new MLA era, the ultimate goal remains unchanged: to produce competent, adaptable and patient-focused doctors who can provide safe, effective care across diverse clinical contexts. When approached collaboratively, the MLA content map offers a promising pathway to achieving this objective, provided that medical schools embrace a truly innovative and collaborative approach to curriculum design.

## CRediT authorship contribution statement

**Isobel Walker:** Writing – review & editing, Writing – original draft, Conceptualization. **Rakesh Patel:** Writing – review & editing, Writing – original draft, Supervision, Conceptualization.

## Declaration of competing interest

The authors declare that they have no known competing financial interests or personal relationships that could have appeared to influence the work reported in this paper.
